# Research on solid shell element based on penalty method

**DOI:** 10.1016/j.mex.2022.101674

**Published:** 2022-03-25

**Authors:** Liu Xintao, Wei Yongtao

**Affiliations:** School of Architecture and Environment, Sichuan University, China

**Keywords:** Solid shell element, Isoparametric element, Penalty factor, Nonlinear analysis

## Abstract

Since the angle degrees of freedom do not have the vector property, it is critical to handle this for nonlinear geometrical behavior. To simulate the nonlinear geometrical behavior of shell structure efficiently, we developed a solid‐shell element type without drilling degrees of freedom. In this paper, the element satisfies the assumption of the straight normal of the shell and we established penalty functions in the thickness direction of the shell. The formulations of equivalent node force and tangent stiffness obtained by 3D isoparametric element theory can be expressed by matrix forms clearly. The constitutive relationship in the global system is derived from the local constitutive matrix according to the tensor coordinate transform. Finally, taking a series of classic flat plate problems and curved shell problems as examples, the solid shell element using the penalty method is compared with the solid shell element using the artificial stiffness method and other shell elements. The results show that the solid element proposed in this paper can effectively eliminate shear self-locking after using the reduced integral scheme and obtain satisfactory accuracy for thin shells and medium-thickness

• A type of solid shell element without rotational degrees of freedom is introduced using the penalty method.
• A suitable penalty factor of this element is determined through numerical experiments.
• This element is more efficient than the traditional shell elements and can be programmed with less time.

## Graphical abstract

**Figure:**
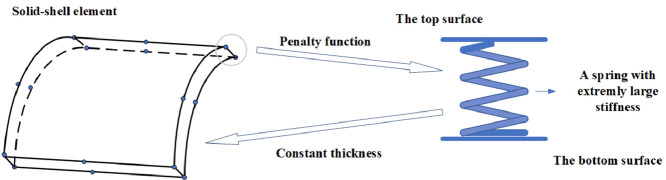

Specifications table

**Table:** 

Subject area:	Engineering
More specific subject area:	*Describe narrower subject area*
Method name:	*A solid shell element with penalty function*
Name and reference of original method:	*None*
Resource availability:	*None*

## *Method details

 

## Introduction

Shell is a very widely used engineering structure. Choosing a reliable element is a prerequisite for obtaining accurate results and solving engineering problems. Since a finite rotation angle is not a vector, it is particularly critical to deal with the degrees of freedom of rotation in a large-deformed shell element. At present, there are three main types of geometrically nonlinear finite element formulations for shells, including folded plate elements composed of flat bending elements and plane stress elements, degenerated two-dimensional elements based on shell theory, and solid shell elements derived from three-dimensional solid elements [1].

The advantage of the folded plate element is that the form of the formulation is relatively simple. However, when this method is used to simulate a shell structure with a smooth surface, the mesh requires high quality or the problem of inconsistent deformation may occur [2]. In particular, this method does not solve the non-vector problem of limited rotation angle under large deformation, and the result obtained under the non-linear situation is quite different from the actual one.

The degenerated two-dimensional shell element is currently the most widely used method. Simo et al. [3] proposed the use of 2D elements to simulate the shell structure based on the shell theory; Bathe and Dvorkin [4], [5], [6], [7], [8] adopted Mixed Interpolation of Tensorial Components (MITC) for the shear strain component, which better solved the shear self-locking phenomenon under large deformation; Arciniega and Reddy [9] deduced the MITC element formula based on the tensor expression under the curve coordinates; Chapelle and Bathe [10,11]. Summarized the general process of the method and explained it with a series of numerical experiments.

The third method is based on three-dimensional solid shell elements. Hauptmann and Schweizerhof [12] proposed the "solid-shell element" and compared it with the degenerate shell element; Klinkel et al. [13] considered the strain in the thickness direction, and designed it according to the 3D nonlinear constitutive equation and a quadrilateral shell element was presented; Tan and Vu-Quoc [14] proposed a calculation formula for the shell under large deformation of nonlinear materials. Zheng Shiyan [15] studied nonlinear composite solid shell elements and modified the element stiffness matrix to ensure continuous stress distribution in the thickness direction of the structure. Hajlaoui et al. [16], [17], [18] used a modified first-order solid shell element formulation to investigate buckling behaviors of functionally graded carbon nanotube-reinforced composites shells and analyzed the static behavior by four different types of reinforcement, which eliminated the need for using the shear correction factor.

Unlike solid shell elements, MITC elements need to use curve tensor analysis to deal with the non-superposition of the angle degrees of freedom. They need to introduce additional interpolation modes to eliminate shear locking, making the element formulation extraordinarily cumbersome and challenging to apply to material nonlinearity. In addition, when there is a discontinuous normal transition on the structure's surface, more processing is required to generate a suitable shell mesh. Compared with the MITC method, the solid shell element can avoid the non-vector of the rotational degrees of freedom naturally. Secondly, the solid shell element has a wide range of thickness (from extremely thin to a specific thickness) when simulating the shell, and the shell thickness is not limited to a constant within the element. Finally, the formula is more straightforward, and the related program is easier to write.

This paper proposes a solid shell element that does not include the angle degree of freedom. The adopted isoparametric interpolation mode makes the element automatically meet the straight normal assumption; in order to eliminate the singularity of the global stiffness matrix that may be caused by the generalized plane stress assumption in the shell theory, a method of introducing the assumption of constant normal length through a penalty function is proposed, and contrast with the artificial stiffness method [19]. Numerical examples show that for thin and medium-thick shells, the reduced integration scheme can effectively eliminate the shear self-locking of the solid element and obtain satisfactory accuracy.

## Shell element mode

### Basic assumptions of the element

The shell theory is based on three basic assumptions. One is the assumption of straight normal, that is, the normal before deformation is still normal (for thin shells) or straight line (for medium thick shells) after deformation; the second is the assumption of generalized plane stress state in the shell coordinate system, ignoring the normal stress; Finally, it is assumed that the thickness of the shell, that is, the length of the normal is always constant. The degenerate two-dimensional shell element is directly constructed based on the shell theory, so these three assumptions are automatically satisfied. The solid shell element needs to introduce the above assumptions into the finite element formulation of the solid element.

### Interpolation mode

**Fig. 1:**
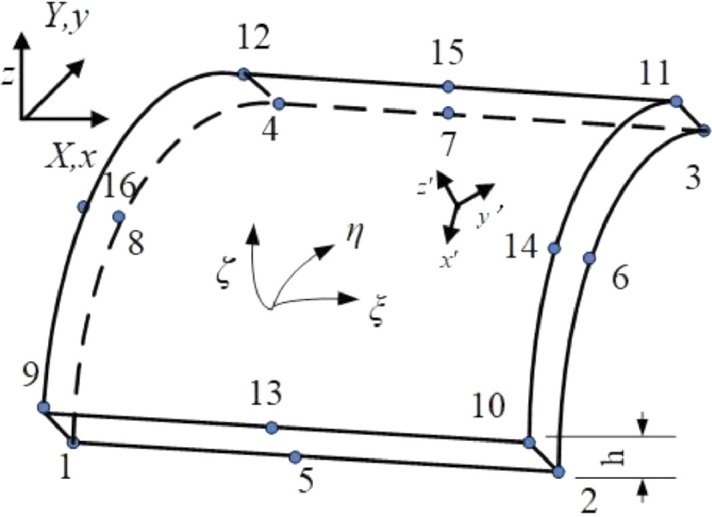
Quadrilateral shell element.

As shown in Fig. 1, for the shell element, *ξ, η* are the local coordinates in the plane, and *ζ* is the local coordinates in the thickness direction. Moreover $X_{a}$,$X_{a+s}$,$u_{a}$, $u_{a+s}$ are coordinates and displacements of the node pairs on the top or bottom of the element, such as node one and node nine.

The shell element uses isoparametric interpolation.

(1) $$\begin{array}{cc} X & =\sum_{a=1}^{2s}N_{a}\left(\xi ,\eta ,\zeta \right)X_{a}\\ & =\sum_{a=1}^{s}\left[\frac{1-\zeta }{2}{\bar{N}}_{a}\left(\xi ,\eta \right)X_{a}+\frac{1+\zeta }{2}{\bar{N}}_{a}\left(\xi ,\eta \right)X_{a+s}\right] \end{array}$$

(2) $$u=\sum_{a=1}^{2s}N_{a}\left(\xi ,\eta ,\zeta \right)u_{a}$$

In the formula, ${\bar{N}}_{a}\left(\xi ,\eta \right)$ is the two-dimensional interpolation basis function in the element surface.

For the material point *A* in the local coordinate which on the connecting line of the node pairs a and a+s in the initial configuration, using Eq. (1), the position of the point in the initial configuration is.

(3) $$X_{A}=0.5\left(1-\bar{\zeta }\right)X_{a}+0.5\left(1+\bar{\zeta }\right)X_{a+s}$$

The position of point *A* in the current configuration is.

(4) $$\begin{array}{c} x_{A}=X_{A}+u_{A}=\frac{1-\bar{\zeta }}{2}\left(X_{a}+u_{a}\right)+\frac{1+\bar{\zeta }}{2}\left(X_{a+s}+u_{a+s}\right)\\ =\frac{1-\bar{\zeta }}{2}x_{a}+\frac{1+\bar{\zeta }}{2}x_{a+s} \end{array}$$

Eq. (3) shows that the point on the connection line between node a and a+s before the deformation is still on the connection line of the pair of nodes after the deformation. The connection line of the pair of nodes before and after the deformation is always straight. Based on Eqs. (1) and (2), it can be further proved that the material line with local coordinates in the initial configuration remains a straight line after deformation.

### Assumptions of generalized plane stress

The stress state in the shell is assumed to be the generalized plane stress state. That is, the normal stress of the shell is considered to be zero.

Assuming $x^{\prime }y^{\prime }z^{\prime }$ be the shell coordinate system at the integration point, and its unit basis vectors${e^{\prime }}_{1}$,${e^{\prime }}_{2}$,${e^{\prime }}_{3}$ are constructed as follows.

(5) $$\begin{array}{cc} {e^{\prime }}_{1} & =\left(\frac{\partial x}{\partial \xi },\frac{\partial y}{\partial \xi },\frac{\partial z}{\partial \xi }\right)\\ {e^{\prime }}_{2} & =\left(\frac{\partial x}{\partial \xi },\frac{\partial y}{\partial \xi },\frac{\partial z}{\partial \xi }\right)\times \left(\frac{\partial x}{\partial \eta },\frac{\partial y}{\partial \eta },\frac{\partial z}{\partial \eta }\right)\\ {e^{\prime }}_{2} & ={e^{\prime }}_{3}\times {e^{\prime }}_{1} \end{array}$$

The conversion matrix θ between the shell coordinate system and the global coordinate system and the transformation relationship between the strain tensor and the stress tensor can be obtained.

(6) $$\theta =\left[\begin{array}{ccc} {e^{\prime }}_{1} & {e^{\prime }}_{2} & {e^{\prime }}_{3} \end{array}\right]$$

(7) $${ɛ}^{\prime }=\theta^{T}ɛ\theta ,\sigma^{\prime }=\theta^{T}\sigma \theta$$

Here σ,ɛ,$\sigma^{\prime }$, ${ɛ}^{\prime }$ are the stress and strain tensors in the global and local coordinate systems, respectively.

The generalized plane stress relationship in the local coordinate system is.

(8) $${\sigma^{\prime }}_{ij}=\left(D_{ijkl}-D_{ij33}D_{33kl}/D_{3333}\right){{ɛ}^{\prime }}_{kl}={\bar{D}}_{ijkl}^{\prime }{{ɛ}^{\prime }}_{kl}$$

where $D_{ijkl}$ is the fourth-order elastic constitutive tensor. For isotropic linear elastic bodies, the matrix form of the above formula is as follows.

(9) $$\left[\begin{array}{c} {\sigma^{\prime }}_{x}\\ {\sigma^{\prime }}_{y}\\ {\sigma^{\prime }}_{z}\\ {\sigma^{\prime }}_{xy}\\ {\sigma^{\prime }}_{yz}\\ {\sigma^{\prime }}_{xz} \end{array}\right]=\frac{E}{1-\nu^{2}}\left[\begin{array}{cccccc} 1 & v & & & & \\ v & 1 & & & & \\ & & 0 & & & \\ & & & \frac{1-\nu }{2} & & \\ & & & & \frac{1-\nu }{2k} & \\ & & & & & \frac{1-\nu }{2k} \end{array}\right]\left[\begin{array}{c} {\varepsilon^{\prime }}_{x}\\ {\varepsilon^{\prime }}_{y}\\ {\varepsilon^{\prime }}_{z}\\ 2{\varepsilon^{\prime }}_{xy}\\ 2{\varepsilon^{\prime }}_{yz}\\ 2{\varepsilon^{\prime }}_{zx} \end{array}\right]$$

where k=1.2 is the shear correction coefficient. When a shell element is a plane, the overall stiffness matrix will be singular because the third main diagonal element of the elastic matrix is 0. Ref. [19] introduces artificial stiffness *q* on the third main diagonal element to prevent the stiffness matrix of the element from being singular.

(10) $$q=0.1\frac{h_{e}^{2}}{l_{e}^{2}}\frac{E}{1-v^{2}}$$

where the characteristic thickness of the element $h_{e}$ can be taken as the mean value of the thickness at the node pairs; the characteristic length $l_{e}$ can be taken as the mean value of the side length of the element.

Convert the strain tensor ɛ in the global coordinate system at the integration point to the shell coordinate system by ${ɛ}^{\prime }=\theta^{T}ɛ\theta$, and then according to $\sigma^{\prime }=\bar{D}:{ɛ}^{\prime }$, the stress tensor σ corresponding in the global coordinate system can be attained. The elastic constitutive tensor ${\bar{D}}_{ijkl}$ in the global coordinate system can be obtained from ${{\bar{D}}^{\prime }}_{ijkl}$ by tensor transformation, and numerical methods can also determine it. Take ${\bar{D}}_{ij11}$ as an example, use ${\tilde{ɛ}}_{11}={ɛ}_{11}+10^{-4}$ to establish the strain tensor $\tilde{ɛ}$, so that the stress tensor $\tilde{\sigma }$ can be derived from in the global coordinate system, then ${\bar{D}}_{ij11}=\left({\tilde{\sigma }}_{ij}-\sigma_{ij}\right)\times 10^{4}$.

## A measure of maintaining normal length

The disadvantage of the above method is that it is not easy to determine a reasonable value of artificial stiffness. For this reason, this paper proposes introducing the assumption of the constant length of normal in the shell theory to eliminate the singularity of the total stiffness matrix.

For the pair of node a and nodea+s, the corresponding constraint equation for the constant length of the normal line is.

(11) $$\begin{array}{c} f_{a}=h_{a}-H_{a}=0\\ h_{a}=\sqrt{{\left(x_{a+s}-x_{a}\right)}^{T}\cdot (x_{a+s}-x_{a})}\\ H_{a}=\sqrt{{\left(X_{a+s}-X_{a}\right)}^{T}\cdot (X_{a+s}-X_{a})} \end{array}$$

where $H_{a}$ and $h_{a}$ are the initial length and the deformed length of the normal at the node a and node a+s, respectively.

Introduce the constraint in the form of a penalty function to define the strain energy.

(12) $$W=\frac{1}{2}Cf_{a}^{2}$$

The penalty factor C is taken as $E\tilde{A}/H$, where $\tilde{A}$ is the area of the shell element divided by the number of node pairs, and *H* is the average thickness of the element. The essence, as mentioned above, is to introduce a spring with high stiffness between each node pair to achieve the requirement of approximately satisfying the constraint conditions.

Strain energy W variation is equal to virtual work of internal force.

(13) $$\delta W=Cf_{a}\delta f_{a}=\frac{Cf_{a}}{h_{a}}{\left[\begin{array}{c} x_{a}-x_{a+s}\\ x_{a+s}-x_{a} \end{array}\right]}^{T}\left[\begin{array}{c} \delta u_{a}\\ \delta u_{a+s} \end{array}\right]$$

The node equivalent effectiveness of the internal force $F_{a}^{C}$ corresponding to this constraint is.

(14) $$F_{a}^{C}=\frac{Cf_{a}}{h_{a}}\left[\begin{array}{c} x_{a}-x_{a+s}\\ x_{a+s}-x_{a} \end{array}\right]=C\left(1-\frac{H_{a}}{h_{a}}\right)\left[\begin{array}{c} x_{a}-x_{a+s}\\ y_{a}-y_{a+s}\\ z_{a}-z_{a+s}\\ x_{a+s}-x_{a}\\ y_{a+s}-y_{a}\\ z_{a+s}-z_{a} \end{array}\right]$$

denote $u_{a}^{C}={\left[\begin{array}{cc} u_{a}^{T} & u_{a+s}^{T} \end{array}\right]}^{T}$

(15) $$\frac{d}{du_{a}^{C}}\left(\frac{1}{h_{a}}\right)=-\frac{1}{h_{a}^{3}}\left[\begin{array}{c} x_{a}-x_{a+s}\\ y_{a}-y_{a+s}\\ z_{a}-z_{a+s}\\ x_{a+s}-x_{a}\\ y_{a+s}-y_{a}\\ z_{a+s}-z_{a} \end{array}\right]$$

The corresponding tangent stiffness matrix.

(16) $$\begin{array}{cc} K_{a}^{C} & =\frac{dF_{a}^{C}}{du_{a}^{C}}=\frac{CH_{a}}{h_{a}^{3}}\left[\begin{array}{c} x_{a}-x_{a+s}\\ y_{a}-y_{a+s}\\ z_{a}-z_{a+s}\\ x_{a+s}-x_{a}\\ y_{a+s}-y_{a}\\ z_{a+s}-z_{a} \end{array}\right]{\left[\begin{array}{c} x_{a}-x_{a+s}\\ y_{a}-y_{a+s}\\ z_{a}-z_{a+s}\\ x_{a+s}-x_{a}\\ y_{a+s}-y_{a}\\ z_{a+s}-z_{a} \end{array}\right]}^{T}\\ & \;+C\left(1-\frac{H_{a}}{h_{a}}\right)\left[\begin{array}{cccccc} 1 & 0 & 0 & -1 & 0 & 0\\ 0 & 1 & 0 & 0 & -1 & 0\\ 0 & 0 & 1 & 0 & 0 & -1\\ -1 & 0 & 0 & 1 & 0 & 0\\ 0 & -1 & 0 & 0 & 1 & 0\\ 0 & 0 & -1 & 0 & 0 & 1 \end{array}\right] \end{array}$$

It should be noticed that, compared with the artificial stiffness method, this method can strictly guarantee the generalized plane stress assumption, but the tangent stiffness matrix derived from the penalty method is also nonlinear under small deformations.

## Numerical example

### Static load on a plate

The side length of the elastic square plate is 1000 mm, the elastic modulus *E* = 2e5 MPa, and the Poisson's ratio v=0.3. The non-dimensional deflection $w_{\max}D/FL^{2}$ at the centre of the plate when subjected to concentrated or uniform loads under different thicknesses t and different constraint conditions are respectively considered, and the results of ANSYS SHELL281 element are compared. D is the bending stiffness of the board.

Table 1 shows the deflection results of 100 N concentrated force on the centre when the four sides are simply supported. among them, the penalty method refers to using the penalty function to introduce the invariable normal length constraint to eliminate the singularity of the total rigidity. The results obtained by both the artificial stiffness method and the penalty method agree with the calculation results of SHELL281 and follow the analytical solution. Therefore, subsequent experiments will only give numerical calculation results based on the penalty method.

**Table 1:** Simply support plate subjected to concentrate force.

Mesh	Small deflection			Large deflection		
	Artificial stiffness	Penalty method	SHELL281	Artificial stiffness	Penalty method	SHELL281
	Thickness = 1 mm					
4 × 4	1.140e-2	1.139e-2	1.160e-2	1.532e-3	1.472e-3	1.474e-3
8 × 8	1.158e-2	1.157e-2	1.158e-2	1.532e-3	1.531e-3	1.532e-3
	Thickness = 10 mm					
4 × 4	1.171e-2	1.167e-2	1.167e-2	1.171e-2	1.167e-2	1.167e-2
8 × 8	1.173e-2	1.169e-2	1.169e-2	1.173e-2	1.169e-2	1.169e-2
Theriacal result:	1.160 × 10^–2^					

To further investigate the situation of the plate at the fixed-support boundary angle and point support, the load is the uniform force or the concentrated force of the resultant force. The calculation results are shown in Tables 2 and 3. The data shows that by controlling the displacement of the top and bottom surfaces of the element at the same time, this element can better simulate the zero corner condition of the fixed boundary without introducing the corner displacement. In addition, the element results are still consistent with the calculation results of SHELL281.

**Table 2:** Fixed plate subjected to distribute force.

Mesh	Small deflection		Large deflection	
	Penalty method	SHELL281	Penalty method	SHELL281
	Thickness = 1 mm			
4 × 4	8.318e-4	8.533e-4	3.568e-3	3.589e-4
8 × 8	1.257e-3	1.257e-3	3.734e-3	3.740e-4
	Thickness = 10 mm			
4 × 4	1.259e-3	1.259e-3	1.259e-3	1.259e-3
8 × 8	1.268e-3	1.268e-3	1.268e-3	1.268e-3

**Table 3:** Corner point simply support plate subjected to concentrate force.

Mesh	Small deflection		Large deflection	
	Penalty method	SHELL281	Penalty method	SHELL281
	Thickness = 1 mm			
4 × 4	3.808e-2	3.905e-2	1.377e-2	1.377e-2
8 × 8	3.819e-2	3.914e-2	1.421e-2	1.420e-2
	Thickness = 10 mm			
4 × 4	3.930e-2	3.930e-2	3.930e-2	3.930e-2
8 × 8	3.931e-2	3.931e-2	3.931e-2	3.931e-2

### Concentrated force on semicircular curved shell

Fig. 3 shows the test of the semicircular curved shell. One end is fixed, the other end is free, and the free end is subjected to concentrated force. The material parameters are the same as in Example 1. The geometric dimensions are shown in Fig. 3. Due to symmetry, the mesh model is half of the actual structure. The grid division mode is shown in Fig. 4. For curved shells with different thicknesses, the deflection of the force point calculated by the element according to the large deformation is shown in Fig. 5 and Fig. 6, respectively. It can be seen from the figure that the quadrilateral solid shell element can always calculate the results consistent with the SHELL281 under various conditions. When the plate and shell thickness is thicker, and the mesh is triangular, compared with the SHELL281 element, the calculated deflection of the force point of the solid shell element is closer to the result of the quadrilateral element when the mesh quality is lower.

### Open toroidal shell under line load

An elastic open thin shell ring is subjected to a pair of transverse shear forces at the end, as shown in Fig. 7. The structural elastic modulus *E* = 2.1e6, the Poisson's ratio is zero, the inner diameter of the ring is R_1_ = 6, the outer diameter R_2_ = 10, the thickness is 0.03, and the radial linear load is 0.8.

The model uses 8 × 36 in-plane meshes with a total of 288 elements. The load-displacement curves of the two points A and B in the vertical direction are shown in Fig. 9. It can be seen that the calculation results of this element are consistent with the calculation results of ANSYS. The element formula used in the literature [1,[20], [21], [22] is relatively complicated, and the matrix form of the solid shell element formula based on the penalty method given in this paper is more concise than them.

Figs. 2 and 8

**Fig. 2:**
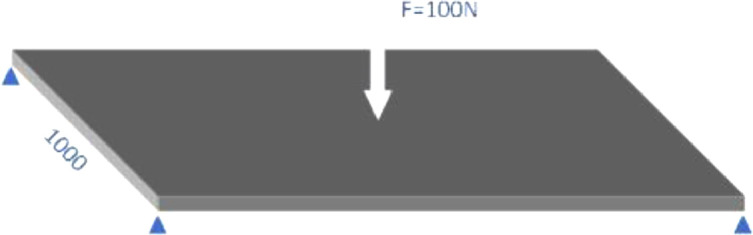
Corner point simply support plate subjected to concentrate force.

**Fig. 3:**
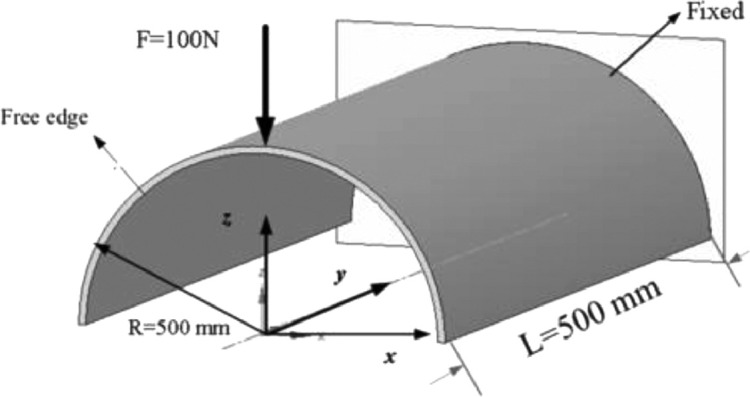
Curved shell subjected concentrate force.

**Fig. 4:**
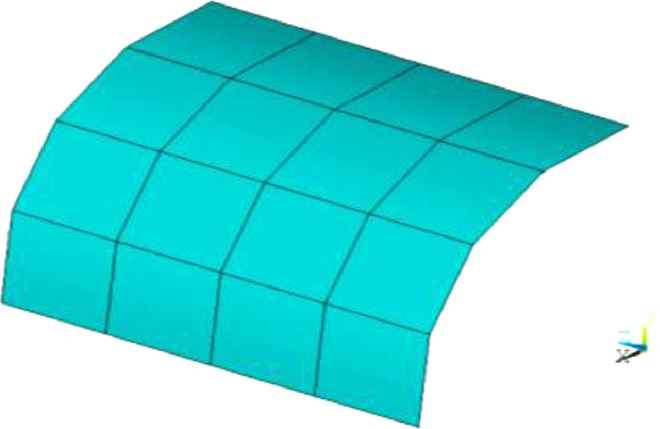
Semicylindrical shell 4 × 4 mesh.

**Fig. 5:**
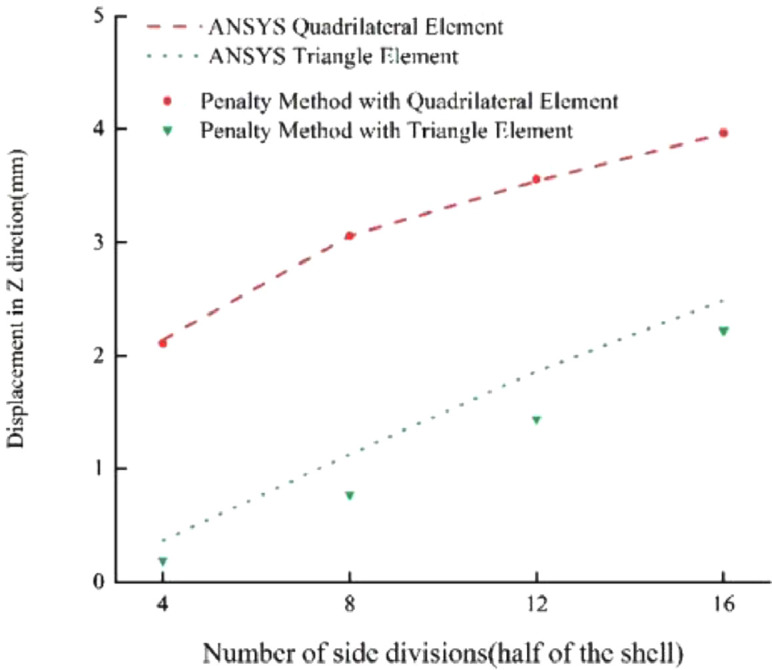
Results for Example 2 (1 mm).

**Fig. 6:**
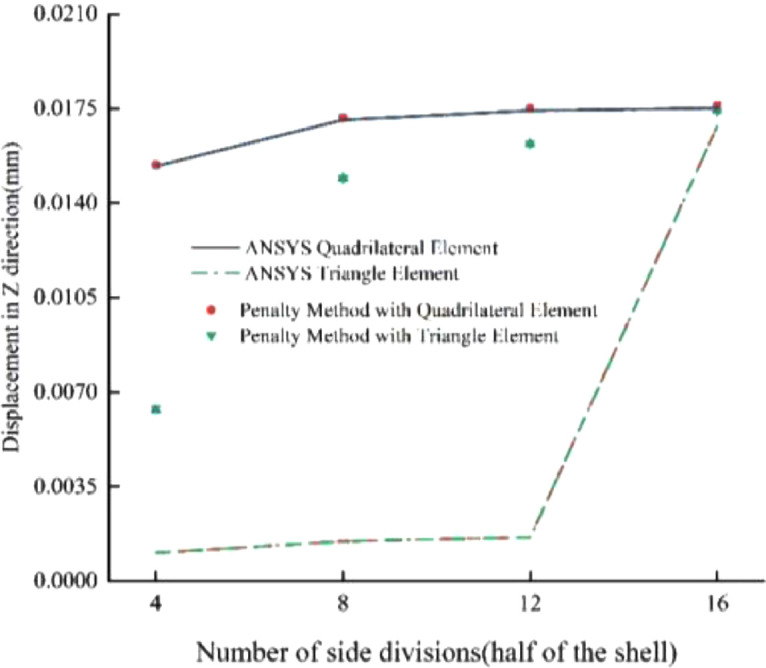
Results for Example 2 (10 mm).

**Fig. 7:**
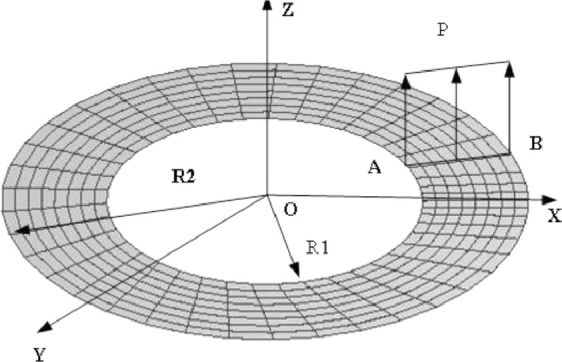
Slit annular plate lifted by a line force.

**Fig. 8:**
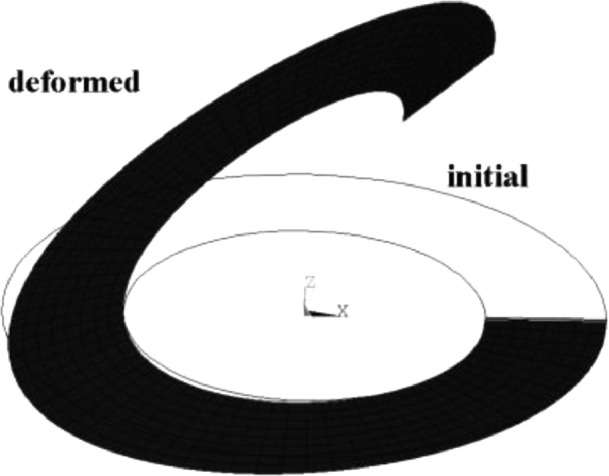
Initial and deformed configuration.

**Fig. 9:**
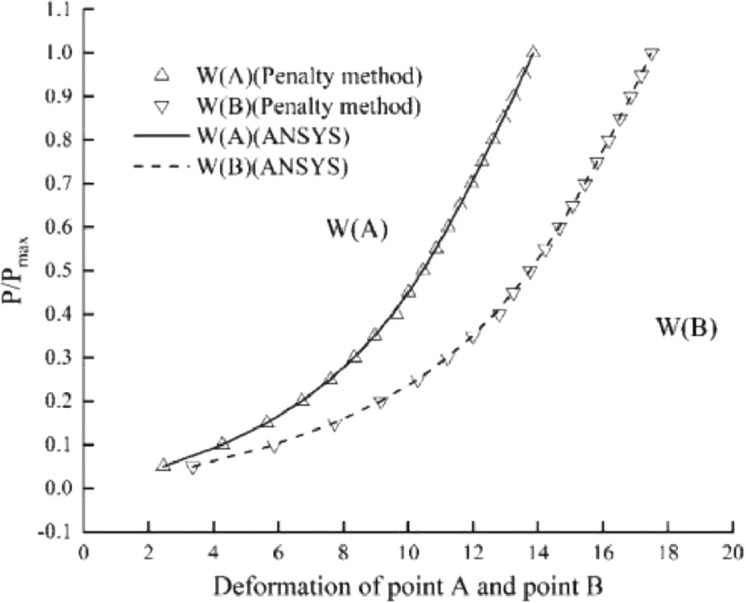
Load-deflection curves for the slit annular plate.

## Conclusion

(1) This paper proposes a solid shell element that does not include the angle degrees of freedom. Through the interpolation mode, the element conforms to the assumption of the straight normal of the shell. Moreover, this paper established a penalty function in the direction of the element normal to ensure that the thickness of the shell element remains unchanged so that the element meets the basic assumptions of the shell.
(2) A series of classic numerical examples show that when the penalty factor is $C=E\tilde{A}/H$, the calculation results of this type of elements agree with the research, proving the method's applicability and accuracy.
(3) The solid shell element can effectively reduce the complexity of the code, thereby speeding up the design of complex engineering programs when used for program design.

## Declaration of Competing Interest

The authors declare that they have no known competing financial interests or personal relationships that could have appeared to influence the work reported in this paper.
